# Don't fall in common science pitfall!

**DOI:** 10.3389/fpls.2014.00536

**Published:** 2014-10-10

**Authors:** Khaled Moustafa

**Affiliations:** Institut National de la Santé et de la Recherche MédicaleCréteil, France

**Keywords:** research misconduct, science ethics, science misbehavior, publication frenzy, publish or perish

The fundamental mission of science in providing knowledge and guidance for solving current and future challenges seems to be changing at accelerated pace, undoubtedly as a result of other economical, technological, and social deep changes. The trend is easily noticeable from an objective and neutral field toward an open, large, and unmerciful business market with many subjective and biased criteria for funding, hiring, promotion, and unscrupulous conducts in many cases. Due to a rubbish “publish-or-perish” mantra, the absence of ethical rules or the ignorance of their existence in a work environment, some scientists weave a kind of intentional or unintentional “tricks” to their way to do or to report science. Questionable conducts ranging from fabricating research data to inappropriate lab notebook records have been reported. While the incidence of research misconducts is unknown precisely at global level (Fanelli, 2009), multiple symptoms and consequences of these drifts are tangible. Funding of research projects for example is entangled in complex competing financial and non-financial interests (Bird and Spier, 2005; Saver, 2012). Scientists are often assimilated to robots; they should be always available, highly productive and permanently at the top of the scale. In career workshops, career professionals do not hesitate to compare candidates to “goods” when they advise or urge candidates to: “sell yourself”! Although the goal is to encourage candidates to draw the attention of stakeholders, the example of “selling” is telling.

Many scientists nowadays are narrowly-specialists with meager knowledge outside of their specialty, their PhD or postdoc's research projects, despite a great facility to gather scientific information and knowledge. In the past, and almost as a rule, most scientists were multidisciplinary experts. It was very common to find a scientist as a doctor, physicist, chemist, biologist, and philosopher at the same time. Although one may argue that there is too much information to grasp in today's multidisciplinary patterns, the availability of extraordinary textual and visual means, such as videos, professional illustrations, easy communication tools, internet, etc., makes it relatively simple and straightforward to understand complex scientific information compared to the past. In other words, the multidisciplinary knowledge today is not proportional to the facilities we enjoy compared to scientists long ago. Moreover, specialization has disadvantageous effects on the cost of science and risk for monopoly and monotony (Casadevall and Fang, 2014).

Another substantial change is a “frenetic” race and high pressure for publication, overweighing in most cases the quantity on quality. To fit with this frenzy, authors are constrained by a harmful “publish-or-perish” refrain to publish every result, as insignificant as it might be, to increase their publication record, sometimes in detriment to reliability, ethic and reproducibility. As a result, the quality of published articles is diluted with redundant, overlapping or messed up findings. To answer these demands, an increasing number of journals are created continuously and many traditional journals are compelled to publish their subsequent issues online, months or sometimes a year before its due date while the corresponding paper versions are considerably delayed, though this is not necessarily a bad thing, as it allows authors to have their papers quickly published and accessible. Other side effects of the “publish-or-perish” mantra include unethical practices and pressure on students with increased numbers of submissions that add further pressure to the existing overload for editors and reviewers (Coimbra, 2009). With the “frenzy” of publish-or-perish, the scientific publication has been transformed into a “fast-food-like publication industry”; in the next years we might see an impact factor, or other dubious metrics, calculated annually, monthly or even daily. Moreover, it seems that if authors cite “renowned scientists” or articles published in “high impact factor” journals, they would increase the “credibility” of their papers! Some snobbish and arrogant scientists tend to cite only articles published in “top” journals, and/or articles published by their friends or people they know (Katchburian, 2008). On the other hand, many papers published by unknown or junior scientists or in journals with low or without impact factors are mostly ignored.

In the past, one-author for the longest work possible was the preponderant rule in the scientific writing field. Now, it is rarely to see one author who writes a manuscript or a book alone, despite unlimited ease to do science and to report it compared to the past. Recently, I have counted up to 200 authors for an article of a few pages. Articles with around 100 authors are more common, particularly in “highly ranked” journals to such an extent readers do not know who did what. Although a long list of authorship could be sometimes justified, particularly in studies involving data at genomic levels (or other complex studies), it is mostly the desire to share the “prestige” of a journal that leads authors to jam on a paper. Moreover, multiple authorships may cause disputes that might result in retraction. Long authorship lists may also compromise the authorship credit because the greater the number of authors, the less credit per author (Hagen, 2014) whereas a full credit for a few papers would be preferred to little or no credit for many (Greene, 2007). Reasons of long authorship lists may include favoritism or political reasons (e.g., inclusion of a well-known person, a previously-included person (but whose results were deleted from the final version of the manuscript), a competitor (to smoothen relationships), or a person who contributed little to the published work but with expectation of future collaborative work.

Paradoxically, while multiple authorship and cooperative research has become the “norm” since some years ago, particularly in applied sciences, the most prestigious award in science (i.e., Nobel Prize) is often granted to one, two or three people only, despite the collaborative characteristic of science (Casadevall and Fang, 2013). The objective for sharing “prestige” leads some authors/journals to add an acknowledgement to say that “*authors X and Y contributed equally to this work*.” However, how would it be possible to contribute “equally” from a practical viewpoint, particularly in writing or performing different experiments, unless by writing exactly the *same number of words* or carrying out exactly *the same experiments*, which is hardly achievable in a scientific work, given that experiments are different and article sections are also of different values and importance? While it should be recognized that “equally-valued” but different types of contributions can be made by two authors, a potential “better” solution might be specifying the contribution of each author “who did what” and let readers judge the importance. Although some journals have already set up such rules, it is still difficult to determine the order of priority of authorship, particularly with the absence of standard about it.

Another “trick” is to read terms such as: *“This is the first time that*…” or “*we describe for the first time that*…” or “*data not shown*”! If data are “not shown,” why to talk about them, then? If they are worthy, they should be shown or provided at least as supplementary material, if not, why to mention it? Moreover, how would it be possible to be sure that it is really the “first time” in a world of ~7 billion people and up, without surveying their thoughts and/or their published books, articles, notes etc., in all languages, all countries, all cities and villages? A more objective prose would be to say “*to the best of our/my knowledge, this is the first study of*…”

The culture of numbers is becoming a misleading mark of “reputation and recognition” (Bauer, 2013). The number of publications in “top journals,” the value of the “impact factor,” the number of citations, the number of patients treated, the number and amount of grants obtained, the number of students trained, the number of conferences attended, the number of “followers,” and “followed” in a social network, the number of “like” or “dislike” in another network, the number of comments on a topic or article, the number of virtual contacts or “friends,” the number of hits, views or downloads etc., became “distorted measures for reputation. There is also an irrational course for classifying everything around us, in every aspect of the academic and non-academic life. From “top” journals to “top” universities, almost everything between is ranked, typically for profit purposes, which would be worse than publish or perish (Kiegle, 2007). Moreover, social networks obviously skew the culture of numbers and quantity mentioned above where “virtual” friends and family members can mislead the virtual impact or reputation of a member of their circle by sharing each other's item in a snow-ball effect.

Another pitfall is the trend toward big business of scientific journals and their publishers. Open access is now an option or compulsory in many journals, but this may favor richer labs and the money paid for this typically comes from the labs and not the institutions whose libraries would otherwise have to pay for subscriptions. Open access journals have added a value to the publication world, but in most cases they are still costly. Open access publishers should reduce their publication cost, since the overall costs have been considerably reduced by saving the costs of paper, printing and shipment.

Additionally, with the increasing number of publications, there is an alarming trend toward low quality peer-reviewing and editing which is often done by less experienced academics. At the same time, publishers are highly profitable. To rectify this issue, reviewers and editors should be remunerated for their efforts to provide quality feedback. However, remuneration of editors and reviewers may raise other problems. That is, only rich publishers will be able to remunerate editors and reviewers, and small publishers or individual journals would be convicted to disappear. Rich publishers may also increase their subscription or their open access fees to cover the remuneration of editors and reviewers, creating new pressures on editors, reviewers, and authors with probably some bias and/or conflict of interest in a paid peer-review process.

A best “idealistic” solution in my view would be the option of “no-fee all-free,” where open access should be free for readers and authors as well. But in such a case, someone should pay the costs of the maintenance and the sustainability of the open access. Some rich institutions have recently started to follow this option, but they are still very few and it is not sure if they will continue to do so in the future and for how much time. The future will show if this system will be viable or not, since some journals have started free, but their publishers now are among the richest publishers over there, due to expensive fees applied after no-fee successful test periods.

To reduce some of the misconducts mentioned above and to make the publication system fairer and transparent, serious efforts are required at different levels. Thesis advisors and research supervisors have great parts of responsibility in making clear to their trainees to behave ethically and to report data as honestly as possible, even if data do not meet the desired expectations (Spier, 2013). Each author and publisher should also make a solemn engagement to maintain the reputation of his profession and the objectivity of his tasks that should be as fair and unbiased as possible. The instauration of respectful and trustful work environment, free from excessive or oppressive pressures, is also a vital incentive for the success of the ethical education (Spier, 2013). Other actions include reducing the price of the open access and the subscription fees, increasing the objectivity and transparency in the peer-review process, reducing the rejection bias (Moustafa, 2014) and make the peer-review as a helpful process to help author publish not as a hole to perish.

Finally, it is worthy to note that some of “the issues” discussed above could be the result of simple ignorance or unintentional errors, particularly from junior trainees or inexperienced scientists who may unconsciously commit some involuntary errors because they simply ignore the ethical context of their work. A distinction between deliberate misconducts, and honest errors should thus be made and explained during educational sessions and mentoring process (Resnik and Stewart, 2012).

## Conflict of interest statement

The author declares that the research was conducted in the absence of any commercial or financial relationships that could be construed as a potential conflict of interest.
